# SLC7A11: the Achilles heel of tumor?

**DOI:** 10.3389/fimmu.2024.1438807

**Published:** 2024-07-08

**Authors:** Yulang Jiang, Mingyu Sun

**Affiliations:** ^1^ Shuguang Hospital Affiliated to Shanghai University of Traditional Chinese Medicine, Shanghai, China; ^2^ Shanghai University of Traditional Chinese Medicine, Shanghai, China; ^3^ Key Laboratory of Liver and Kidney Diseases, Institute of Liver Diseases, Shuguang Hospital Affiliated to Shanghai University of Traditional Chinese Medicine, Shanghai, China

**Keywords:** SLC7A11, redox homeostasis, tumor metabolism, immune, cell death

## Abstract

The non-natriuretic-dependent glutamate/cystine inverse transporter-system Xc- is composed of two protein subunits, SLC7A11 and SLC3A2, with SLC7A11 serving as the primary functional component responsible for cystine uptake and glutathione biosynthesis. SLC7A11 is implicated in tumor development through its regulation of redox homeostasis, amino acid metabolism, modulation of immune function, and induction of programmed cell death, among other processes relevant to tumorigenesis. In this paper, we summarize the structure and biological functions of SLC7A11, and discuss its potential role in tumor therapy, which provides a new direction for precision and personalized treatment of tumors.

## Introduction

Tumors, as abnormal growths in the body, often demonstrate a heightened metabolic state that fuels their relentless expansion. This amplified metabolic activity is crucial for their survival and growth, as they voraciously consume nutrients to maintain their proliferative e momentum. To achieve this, tumors enhance their uptake of essential nutrients, ensuring a steady supply for their rapid cellular division and growth. This altered metabolic behavior is a hallmark of malignancy, reflecting the tumor’s adaptive response to meet its energetic and biosynthetic demands. Consequently, this heightened metabolic rate not only underscores the aggressive nature of the disease but also offers potential therapeutic targets for disrupting tumor growth (1–3). The unique amino acid transporter-Solute Carrier Family 7 Member 11(SLC7A11) has been found to be significantly upregulated in various tumor types, with its expression levels closely associated with tumor cell proliferation, invasion, metastasis, and the tumor microenvironment (4–6). Additionally, SLC7A11 has been linked to resistance to radiation and conventional chemotherapeutic agents (7, 8).Thus, SLC7A11 may serve as a promising biomarker for the diagnosis and prognostication of clinical tumors (9).

The significant abundance of SLC7A11 suggests its potential as a promising target for tumor therapy (10). SLC7A11 plays a crucial role in facilitating the import of cystine into cells for the synthesis of glutathione, which is essential for maintaining intracellular glutathione (GSH) levels and protecting cells from oxidative stress-induced damage (5). This process is intricately linked to the initiation of ferroptosis. The SLC7A11/GSH/GPx4 axis serves as the central defense mechanism against ferroptosis, and downregulating the expression and activity of SLC7A11 has been shown to enhance the sensitivity of tumor cells to ferroptosis (11). Moreover, the elevated intracellular GSH levels induced by SLC7A11 confer inherent resistance to oxidative stress therapy in cells. A novel approach in the realm of cancer treatment involves the use of immunotherapy-activated CD8^+^T cells, which release IFN-γ to enhance tumor cell ferroptosis via PD-L1 inhibition (12, 13). IFN-γ secretion decreases SLC7A11 expression, suggesting that combining SLC7A11 inhibitors with immunotherapy could improve cancer treatment (13, 14). The recent study revealed a significant reliance on NADPH and glucose in tumor cells expressing high levels of SLC7A11, thereby questioning the conventional understanding of SLC7A11 as a promoter of cancer (15). Suppression of glucose uptake in the presence of elevated SLC7A11 expression leads to intracellular disulfide stress, ultimately resulting in cell death (16).

**Figure 1:**
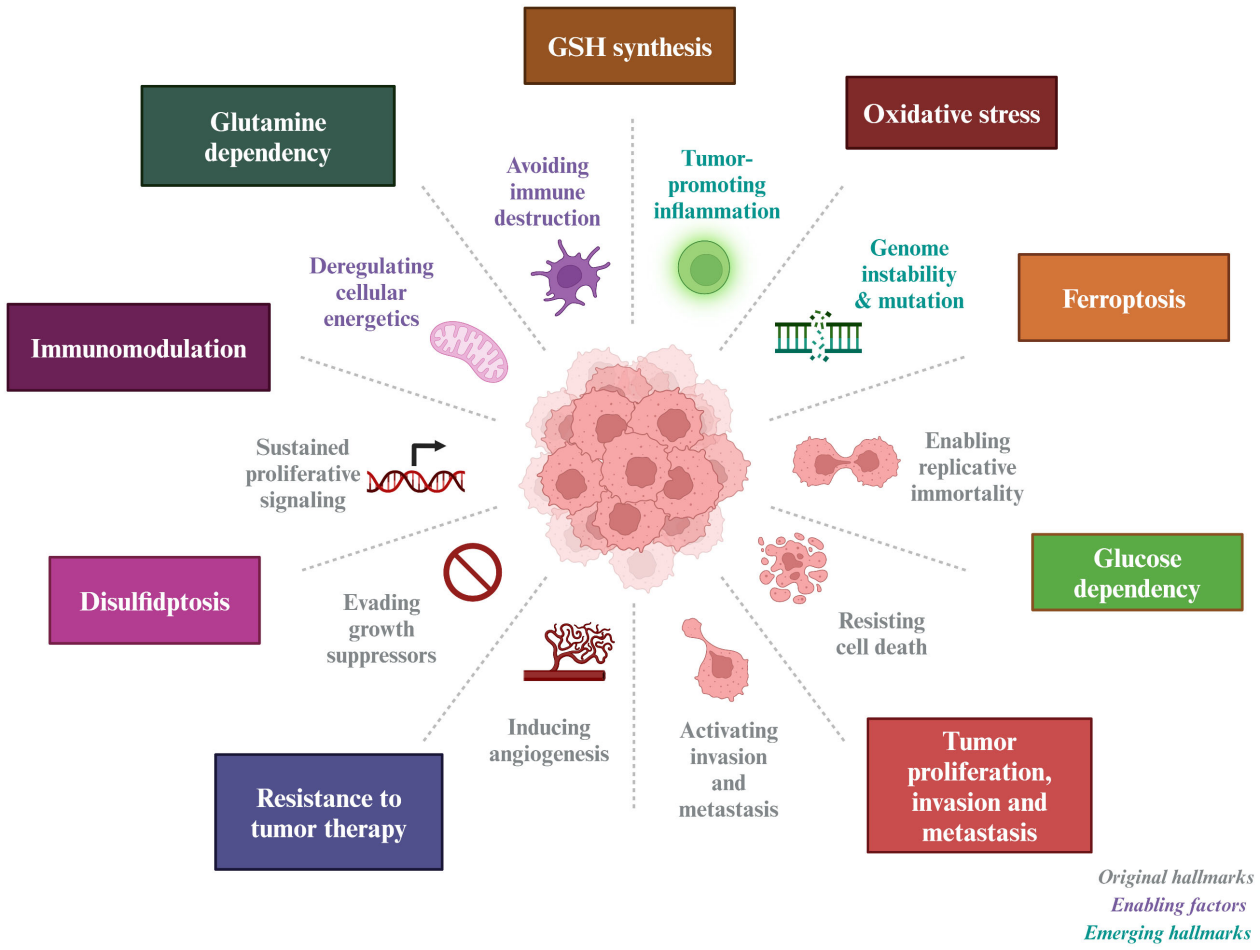
Tumor-based characterization of the interventional role of SLC7A11. SLC7A11 can improve tumor resistance to radiotherapy and inhibit tumor proliferation, invasion and metastasis by affecting GSH synthesis, inducing oxidative stress, causing glucose and glutamine dependence in tumor cells, and inducing ferroptosis and disulfidptosis in tumor cells.

Given the pivotal role of SLC7A11 in cancer treatment, this paper provides an overview of its structure and biological functions, as well as its involvement in oxidative stress, tumor metabolism, immune modulation, and cell death (**Figure 1**).

## Structure and function of SLC7A11

System Xc- is composed of two subunits: the light chain functional subunit SLC7A11, also referred to as xCT, and the heavy chain structural subunit SLC3A2, also known as CD98 or 4F2hc (17, 18).

**Figure 2:**
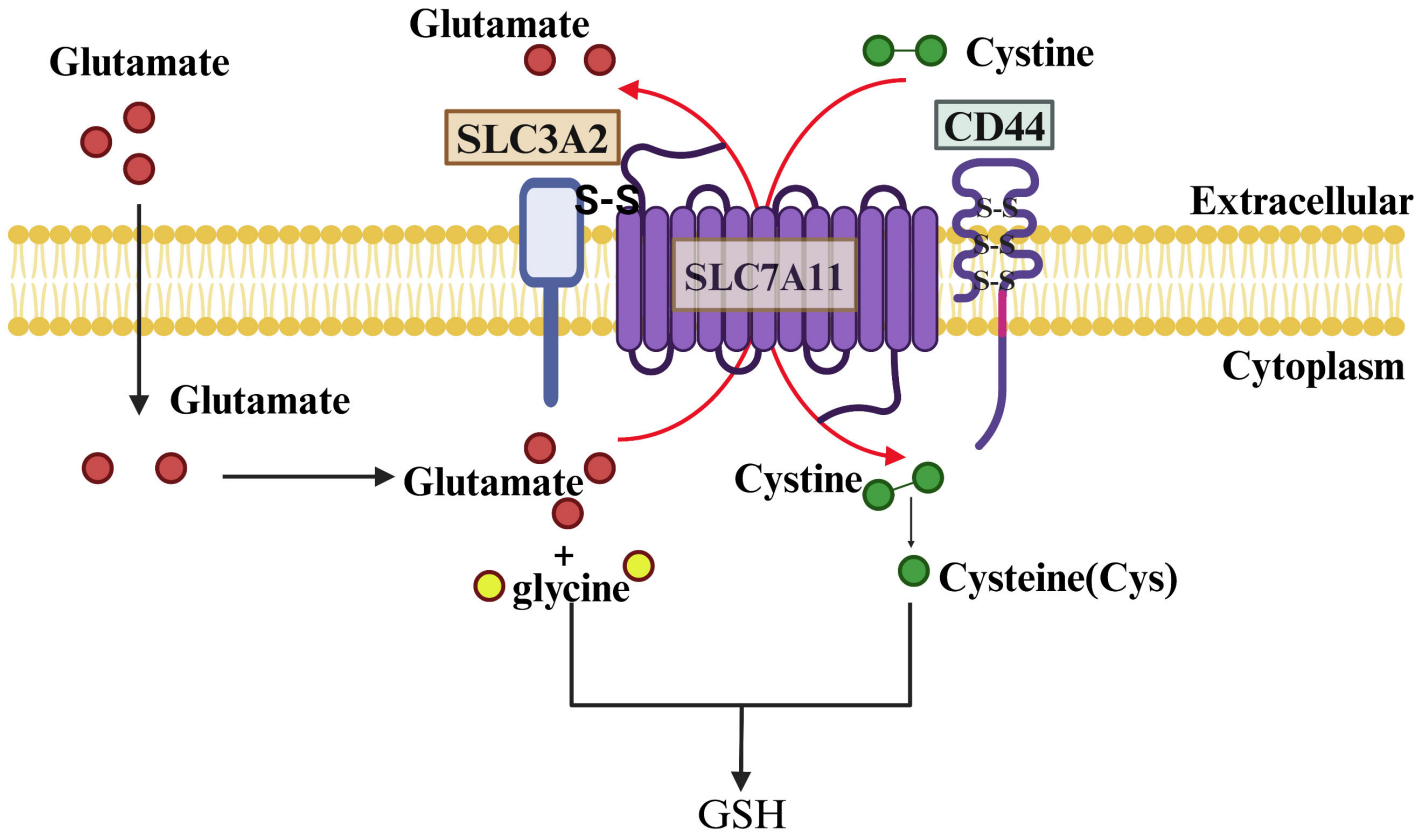
Structure and Function of SLC7A11. SLC7A11 and SLC3A2 collectively constitute the glutamate/cystine reverse transporter, with SLC7A11 being a 12-transmembrane-spanning protein featuring intracellular N- and C-termini, and SLC3A2 being a single-transmembrane-spanning protein with an extracellular N-terminus and an intracellular C-terminus. The two proteins are linked by a disulfide bond and function to facilitate the cellular uptake of cystine and the extrusion of glutamate. Cystine is rapidly reduced to cysteine inside the cell and combined with a molecule of glutamate and glycine to synthesize GSH under the action of GCL and GSS enzymes.

In humans, the gene encoding SLC7A11 is situated on chromosome 4 and comprises 14 exons, resulting in a protein sequence of 502 amino acids. SLC7A11 is a transmembrane protein with 12 transmembrane domains, with both its N- and C-termini located intracellularly (19). This protein is abundantly expressed in various tissues and cells throughout the human body and is evolutionarily conserved among vertebrates. In contrast, SLC3A2 is a type 2 membrane glycoprotein characterized by a single transmembrane structural domain, with its N-terminal end intracellular and its C-terminal end extracellular and heavily glycosylated. SLC7A11 is linked to SLC3A2 through a disulfide bond formed between the conserved residue Cys 158 of SLC7A11 and Cys 109 of SLC3A2 (20). Notably, SLC3A2 serves as the chaperone protein for various members of the light subunits of heterodimeric amino acid transporters (LSHAT) family. Consequently, the determination of substrate specificity in system Xc-is primarily influenced by SLC7A11, while SLC3A2 plays a role in facilitating the transportation of SLC7A11 into the intracellular compartment or potentially enhancing the stability of the SLC7A11 protein (21). Furthermore, CD44 has been identified as capable of interacting with and stabilizing SLC7A11 on the membrane of cancer cells (22) (**Figure 2**).

A variety of amino acid transporters present on the cell surface facilitate the uptake of amino acids by cells, with system Xc- being the main known amino acid transport complex responsible for the transportation of cystine (23). This complex operates by importing cystine into the cell and exporting glutamate out of the cell in a 1:1 ratio, without the need for sodium ions. Once inside the cell, cystine is promptly converted to cysteine, which plays a crucial role as the limiting factor in the synthesis of glutathione (24). Therefore, the expression level and activity of SLC7A11 are the main factors affecting GSH content.

## Expression of SLC7A11 in tumors and prognosis

Since its initial identification, research has revealed that SLC7A11 exhibits a tissue-specific distribution, with varying levels of mRNA abundance across 27 different tissues. The findings indicate that SLC7A11 is most prominently expressed in the brain, followed by the thyroid, stomach, appendix, bladder, and gallbladder, while demonstrating lower expression levels in the kidney, heart, and liver. Given the specific subcellular localization of SLC7A11 and its recognized functional significance, it is not unexpected that a multitude of studies have consistently demonstrated the involvement of SLC7A11 in various neurodegenerative, ocular, and immune disorders (25, 26).

It is noteworthy that SLC7A11 is prominently expressed in numerous tumors and exerts influence on tumor progression, invasion, metastasis, and unfavorable prognosis. Elevated levels of SLC7A11 expression have been demonstrated in a diverse array of tumor types, such as lung, liver, pancreatic, breast, ovarian, prostate, bladder, colorectal, melanoma, and leukemia, in comparison to healthy tissues (9, 27–30). Particularly in oncology patients who exhibit insensitivity to initial therapeutic agents and demonstrate resistance to radiotherapy and chemotherapy interventions (31). **Table 1** lists the expression and prognosis of SLC7A11 in different tumors.

**Table 1:** Expression levels and prognosis of SLC7A11 in different tumors.

Tumor type	expression	Prognosis-related
ACC	UP	poor prognosis
BLCA	unchanged	No significant effects
BRCA	UP	poor prognosis
CESC	UP	No significant effects
CHOL	UP	poor prognosis
COAD	UP	poor prognosis
DLBC	UP	No significant effects
ESCA	UP	No significant effects
GBM	unchanged	No significant effects
HNSC	UP	No significant effects
KICH	UP	No significant effects
KIRC	UP	No significant effects
KIRP	UP	poor prognosis
LAML	UP	poor prognosis
LGG	UP	No significant effects
LIIC	UP	No significant effects
LUAD	UP	No significant effects
LUSC	UP	No significant effects
MESO	UP	poor prognosis
OV	UP	No significant effects
PAAD	UP	poor prognosis
PCPG	UP	No significant effects
PRAD	UP	No significant effects
READ	UP	No significant effects
SARC	UP	poor prognosis
SKCM	unchanged	No significant effects
STAD	UP	No significant effects
TGCT	UP	No significant effects
THCA	unchanged	No significant effects
THYM	unchanged	No significant effects
UCEC	UP	No significant effects
UCS	UP	No significant effects
UVM	UP	poor prognosis

Mechanically, tumor tissues tend to enhance their own antioxidant defenses in response to high levels of oxidative stress by up-regulating SLC7A11 expression, while SLC7A11-mediated synthesis of GSH acts as a defense against the cytotoxic effects of radiotherapy or certain drugs, which further reduces the sensitivity of tumor cells to treatment.

## Role of SLC7A11 in cancer therapy

SLC7A11 expression causes cellular ferroptosis or enhances tumor killing by immune cells by affecting oxidative status or nutrient and energy metabolism in the tumor microenvironment (TME). Here we summarize the potential role of SLC7A11 in cancer.

## SLC7A11 and oxidative stress

Oxidative stress is cellular and tissue damage caused by the production of reactive oxygen species (ROS) in the organism exceeding their removal.

ROS are products of normal physiological activities and are a general term for a class of chemically active molecules and ions with high oxidative activity, which play important roles in cell signaling and tissue homeostasis (32). Essentially, ROS are partially reduced oxygen-containing molecules, including superoxide anion (O_2_^-^), peroxides (H_2_O_2_ and LOOH), and free radicals (HO· and LO·). Mitochondria are the main site of ROS production, of which more than 90% are produced by mitochondria in the normal metabolism and energy supply of the electron transport chain (33). Also, ROS can be produced by cytochrome P450, NADPH oxidase (NOX), xanthine oxidase (XO), and peroxidase in the microsomes (34). Of course, the body also exists a ROS scavenging system to maintain homeostasis, which is mainly categorized into enzymatic antioxidants and non-enzymatic antioxidants.

Non-enzymatic antioxidants scavenge free radicals by interacting directly with them. Such substances include glutathione, vitamin A, vitamin C, vitamin E, and coenzyme Q10. Enzymatic antioxidants, on the other hand, act as antioxidants by catalyzing the degradation of ROS. The main ones are Super Oxide Dismutase(SOD), Trx system and Gpx family (35).

**Figure 3:**
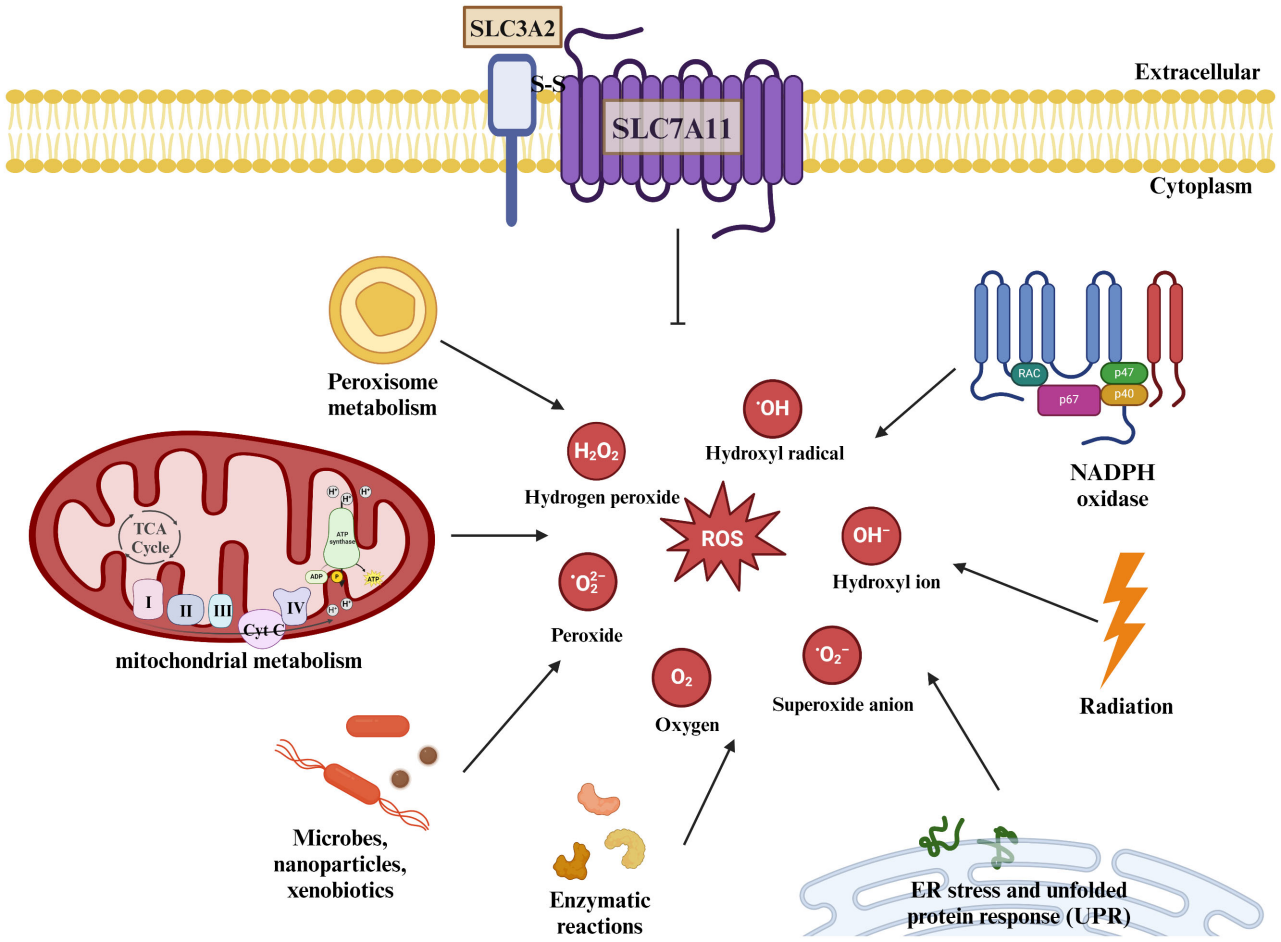
SLC7A11 regulates cellular redox homeostasis. ROS are present intracellularly in a variety of forms: including superoxide anions, peroxides, and oxygen radicals. ROS are present intracellularly in a variety of forms: including superoxide anions, peroxides, and oxygen radicals. Mitochondria produce large amounts of ROS through the electron transport chain, and the metabolism of NADPH and peroxisomes also increases intracellular ROS levels, as do some microorganisms or misfolded abnormal proteins, and studies have shown that radiation therapy also produces some ROS. The human body can scavenge excess ROS under normal physiological conditions through SLC7A11-mediated production of GSH. The body can remove excessive ROS accumulation through SLC7A11-mediated GSH production under normal physiological conditions to maintain redox homeostasis.

The Gpx family of glutathione peroxidases is an evolutionarily highly conserved group of enzymes containing eight main isoenzymes, among which Glutathione Peroxidase 4(GPx4) utilizes GSH as a cofactor to convert lipid hydroperoxides into nontoxic lipids alcohols and reduce free radical accumulation (36). Inhibition of GPx4 can lead to an increase in ROS whereas increased GPx4 expression can lead to a decrease in ROS content, and GPx4 performs this function dependent on GSH (37), which is an important component of the body’s endogenous antioxidant system, and is derived from glutamate, glycine, and cysteine by dehydration condensation, and its intracellular level is affected by the ability of SLC7A11 to transporter cystine (38). Studies have shown that pharmacological inhibition of SLC7A11 or knockdown of SLC7A11 reduces intracellular cysteine concentration, thereby affecting GSH concentration (39). erastin, a selective inhibitor of SLC7A11, inhibits cystine uptake, and salicylsulfonylpyrimidines and glutamate can also reduce intracellular cysteine concentration and deplete intracellular GSH by inhibiting SLC7A11 (40), depleting GSH in cells. thereby inducing cellular oxidative stress and death (**Figure 3**).

In conclusion, SLC7A11 has a pro-survival effect, and SLC7A11-mediated cystine uptake can help cells re-establish redox homeostasis in response to oxidative stress, whereas inhibition of SLC7A11 can lead to depletion of cellular GSH and thus make the cells more sensitive to chemotherapy or radiotherapy.

## SLC7A11 and tumor metabolism

Beyond its well-documented antioxidant functions, SLC7A11 emerges as a pivotal metabolic regulator that profoundly influences intracellular nutrient processing and energy metabolism within cancer cells. A fascinating aspect of SLC7A11’s role in tumorigenesis is its ability to modulate the uptake and conversion of cystine. Cancer cells with elevated levels of SLC7A11 expression exhibit an increased affinity for cystine, which they rapidly convert into cysteine. This biochemical transformation, however, comes at an energetic cost (41).

**Figure 4:**
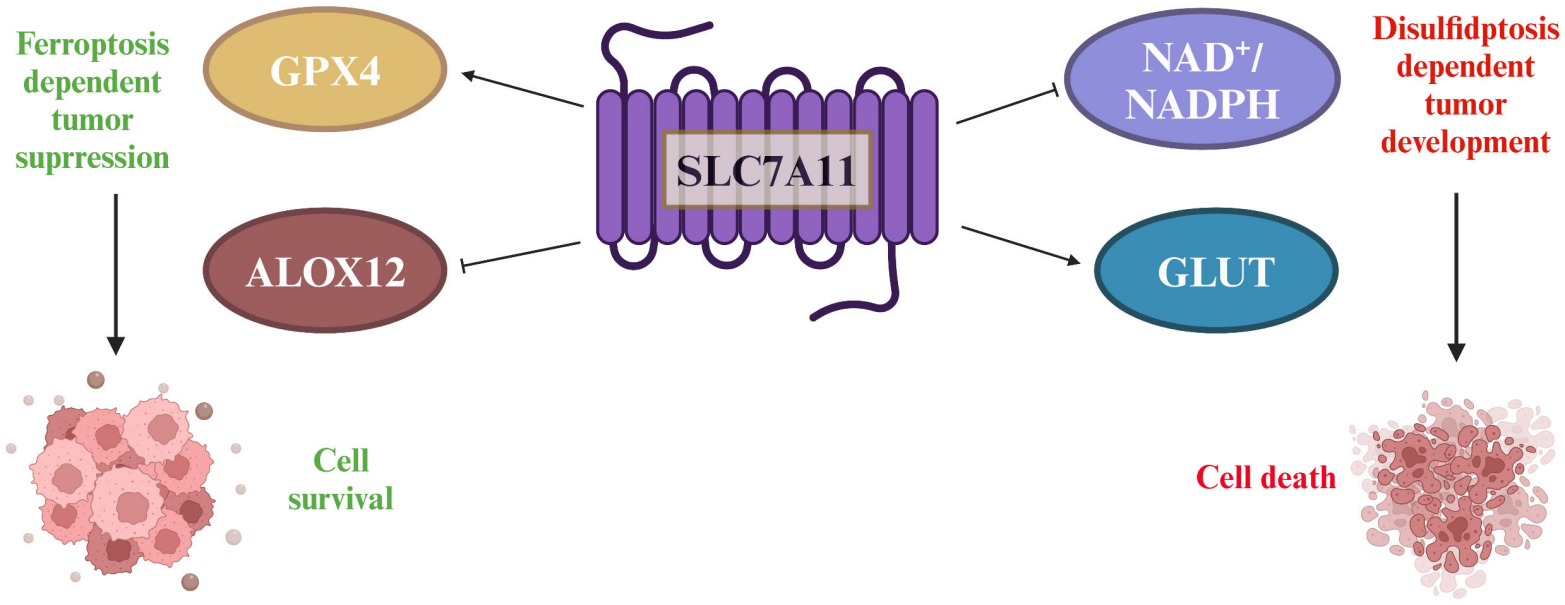
The double-edged sword played by SLC7A11 in tumors. SLC7A11 overexpression in tumors increases cysteine and GSH levels, which are important for reducing lipid peroxides through GPx4. SLC7A11 also suppresses ALOX12, decreasing lipid peroxides and promoting tumor cell survival. However, high SLC7A11 expression leads to cystine accumulation, requiring NADPH from the pentose phosphate pathway to convert it to cysteine. Inhibiting glucose transporter proteins depletes glucose, causing disulfide stress and cell death.

Specifically, the conversion process requires a significant amount of NADPH, a crucial cofactor that primarily originates from the cytoplasmic glucose-pentose phosphate pathway. This pathway, in turn, plays a vital role in generating ribose-5-phosphate for nucleotide synthesis and NADPH for reductive biosynthesis and detoxification of reactive oxygen species. Consequently, cancer cells overexpressing SLC7A11 develop a heightened dependency on both the glucose and pentose phosphate pathways for their survival and proliferation (42). This enhanced reliance on glucose metabolism renders these cells particularly vulnerable to glucose deprivation. In the context of glioblastoma, for instance, glucose starvation can induce cell death more rapidly in cells with high SLC7A11 expression. This vulnerability presents a potential therapeutic window. By simultaneously targeting glucose transporter type 1 (GLUT1), a glucose transporter critical for glucose uptake, and glutathione synthesis, which is intimately linked to NADPH production and reactive oxygen species scavenging, it may be possible to deplete NADPH levels and cause a buildup of reactive oxygen species. This approach could potentially trigger synthetic lethal cell death specifically in cell lines that overexpress SLC7A11 and are thus sensitized to glucose deprivation, offering a promising avenue for targeted cancer therapies (43) (**Figure 4**).

SLC7A11 also affects the nutrient dependence of tumor cells through glutamine backfilling and GLS dependence (43). SLC7A11-mediated glutamate transport may deplete the intracellular glutamate/α-KG pool and activate glutamine catabolism, leading to greater glutamine uptake (44).

## SLC7A11 and immune regulation

Within the tumor microenvironment, interactions involving SLC7A11 between immune cells and tumor cells play a significant role in influencing tumor survival and proliferation. Specifically, cytokines released by immune cells have the potential to impact the expression of SLC7A11 within tumors. For instance, the secretion of interferon gamma (IFN-γ) by CD8+ T cells has been shown to down-regulate the expression of SLC3A2 and SLC7A11 in tumor cells, leading to a disruption in cystine uptake (45). This disruption ultimately promotes lipid peroxidation and iron-induced cell death within the tumor cells (46). Conversely, the interplay of cysteine competition and glutamate secretion among various immune cells, as well as between immune cells and tumor cells, significantly impacts the survival of tumors. Cysteine, a crucial amino acid for T-cell activation, is integral to tumor surveillance and cytotoxicity (12). T cells, lacking the functional SLC7A11 transporter protein and cystathionine beta-synthase enzyme, depend on neutral amino acid transporter proteins to uptake cysteine exported by antigen-presenting cells (APCs) (47).

**Figure 5:**
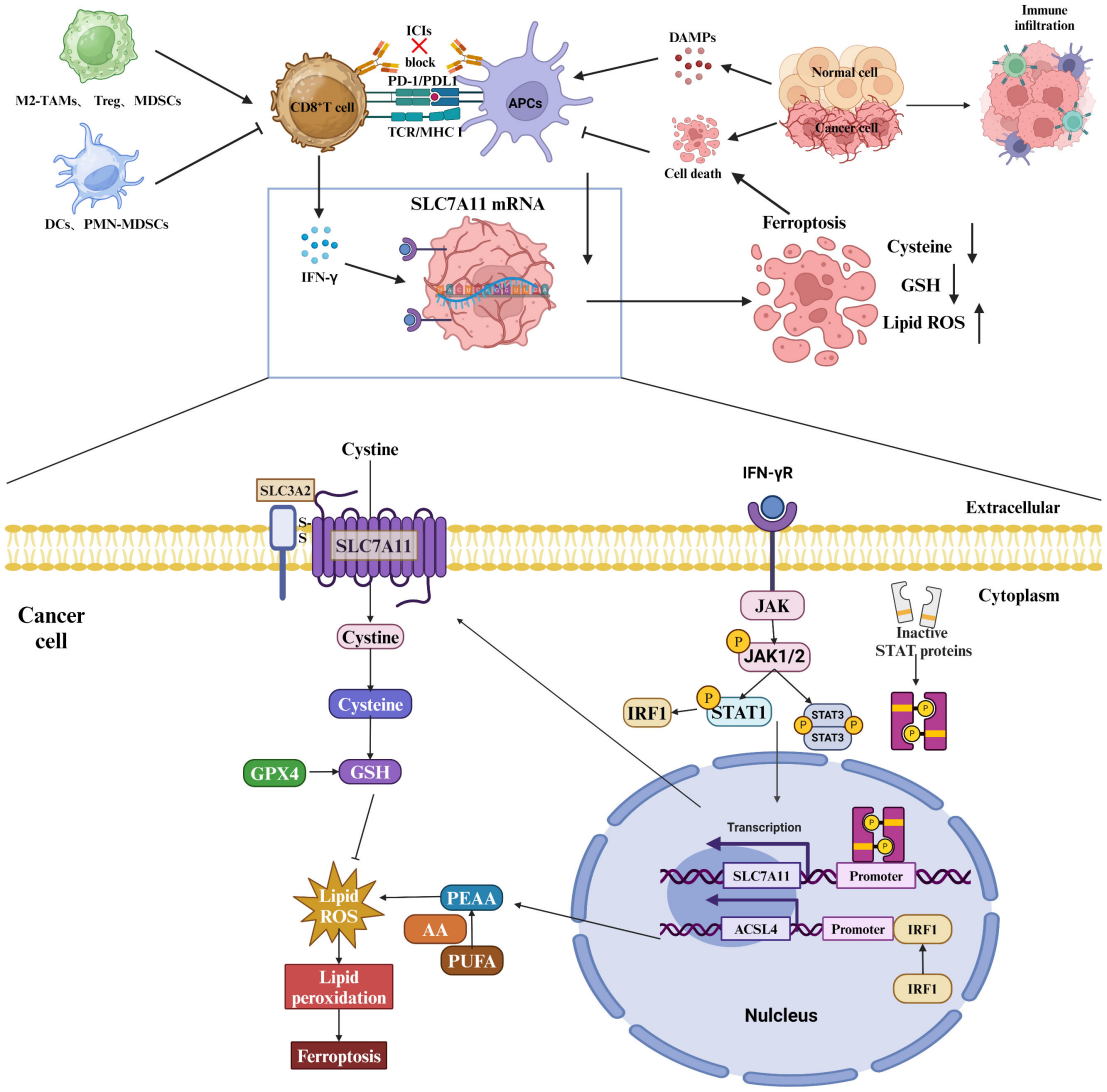
Role of SLC7A11 in regulating the tumor immune microenvironment. Myeloid-derived suppressor cells (MDSCs) express the transporter SLC7A11, which selectively transports cystathionine but does not export cysteine. MDSCs compete for extracellular cystathionine with antigen-presenting cells (APCs), resulting in a decrease in cysteine release from APCs in the presence of MDSCs. This limitation of the extracellular cysteine pool hinders T cell activation-mediated antitumor immunity. In addition, SLC7A11-mediated glutamate release in dendritic cells inhibits metabotropic glutamate receptors, thereby impairing T cell activation. Upregulation of SLC7A11 in glioblastoma (GBM) leads to elevated extracellular glutamate levels, which favors the proliferation, activation, and suppressive capacity of regulatory T (Treg) cells and thus promotes intratumorally immunosuppression. T-cell-induced metabolic alterations can affect the fate of GBM cells via SLC7A11, and tumor metabolism via SLC7A11-mediated dysfunction impairs T cell activation, which further promotes immune evasion. Specifically, IFN-γ affects the mRNA levels of SLC7A11 and ACSL4 through the JAK/STAT signaling pathway.

Myeloid-derived suppressor cells (MDSCs) express the transporter SLC7A11, which selectively transports cystathionine but does not export cysteine. MDSCs compete with APCs for extracellular cystine, leading to a reduction in APC release of cysteine in the presence of MDSCs (45). This limitation of the extracellular pool of cysteine hinders T cell activation-mediated antitumor immunity. Additionally, SLC7A11-mediated glutamate release in dendritic cells inhibits metabotropic glutamate receptors, impairing T cell activation (48). The upregulation of SLC7A11 in glioblastoma (GBM) leads to heightened levels of extracellular glutamate, facilitating the proliferation, activation, and suppressive capabilities of regulatory T (Treg) cells, consequently fostering intratumoral immunosuppression (49). Metabolic alterations induced by T cells can influence the fate of GBM cells through SLC7A11, while tumor metabolism further contributes to immune evasion by impairing T cell activation via SLC7A11-mediated dysfunction (**Figure 5**).

## SLC7A11 and cell death

**Figure 6:**
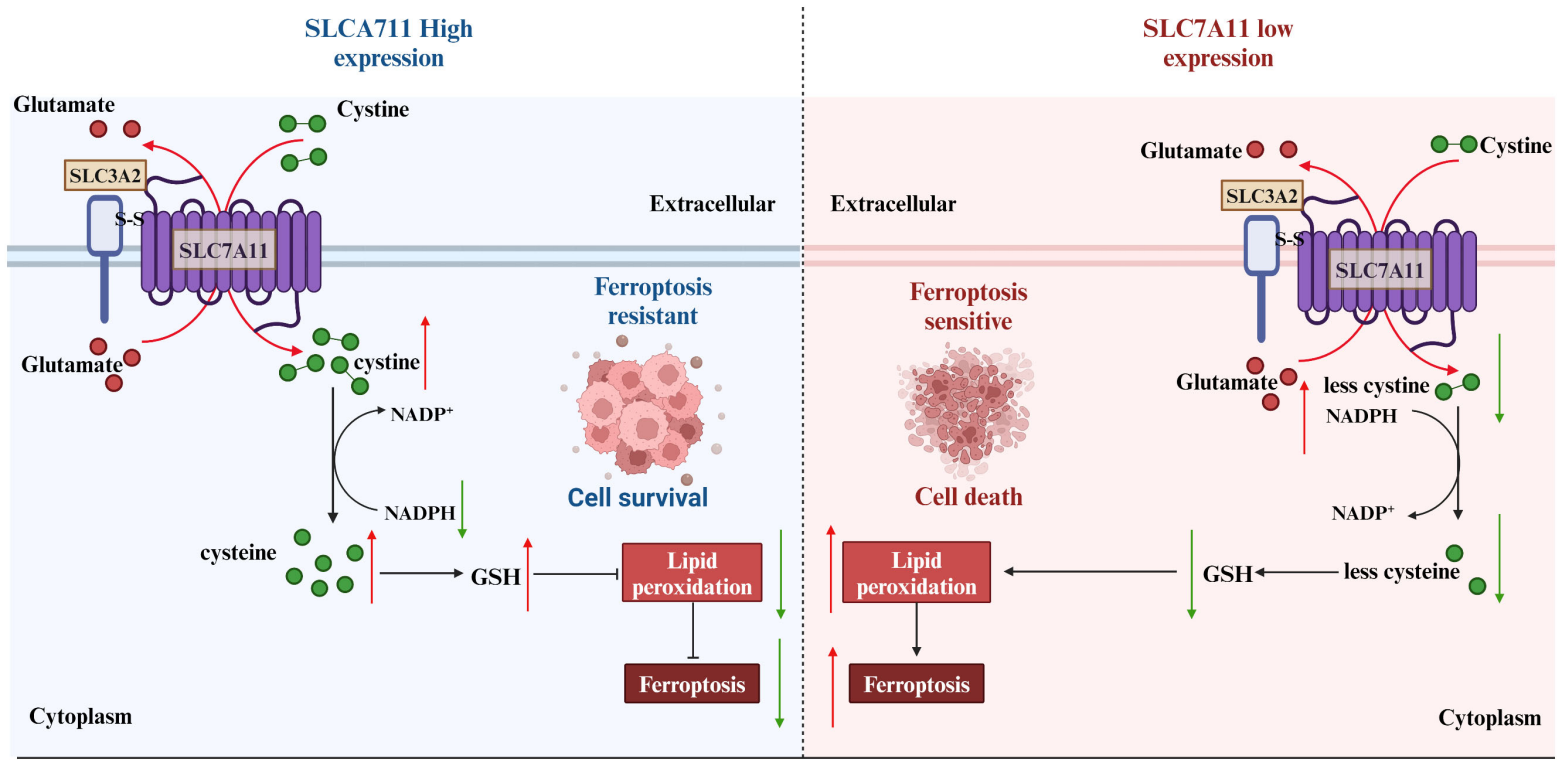
Strategies of SLC7A11 in mediating ferroptosis in the treatment of tumors. The expression levels of SLC7A11 in different cancers were characterized differently. For cells with low SLC7A11 expression, intracellular GSH deprivation leads to diminished lipid peroxide scavenging, resulting in tumor cells that are sensitive to ferroptosis. In contrast, tumor cells with high SLC7A11 expression would be naturally highly resistant to ferroptosis.

In the initial investigations into cell growth conditions, researchers observed that a lack of SLC7A11-mediated cystine transport proved fatal for certain cells (50, 51). Subsequent findings revealed that cells deficient in cystine exhibited diminished levels of intracellular GSH, a key antioxidant, and that supplementation with vitamin E effectively prevented this form of cell death induced by cystine deficiency (52–54). These results imply a strong association between this mode of cell death and oxidative stress. Further research identified this specific form of cell death as ferroptosis (55). Ferroptosis is characterized by the accumulation of lipid peroxidation products resulting from iron metabolism and ROS accumulation (56). The SLC7A11/GSH/GPx4 axis plays a central role in the cell’s defense against ferroptosis, with GPx4 utilizing glutathione (GSH) as a cofactor to reduce toxic lipid peroxides at the plasma membrane, thereby protecting the cell from ferroptosis. SLC7A11 is positioned at the initial stage of the ferroptosis pathway, and inhibitors targeting SLC7A11 are commonly used as inducers of ferroptosis in research studies (57)(**Figure 6**).

**Figure 7:**
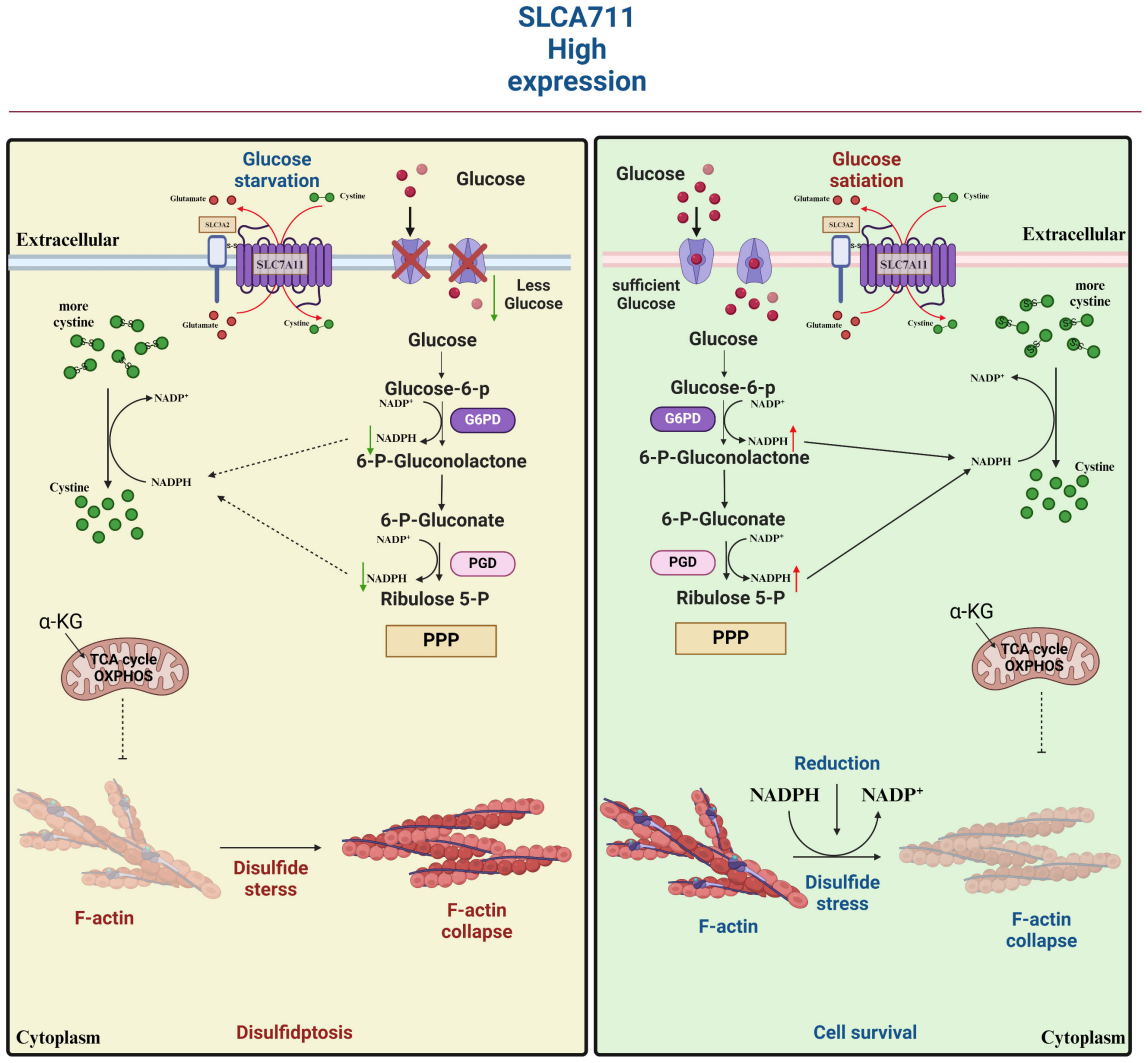
SLC7A11 is highly expressed in tumor cells in response to glucose-dependent induction of disulfidptosis. Disulfidptosis, a unique form of cell death, relies on redox reactions and disulfide bond formation. This process is controlled by cystine uptake and glucose metabolism, with SLC7A11 playing a key role in transporting cystine for glutathione synthesis. However, cystine can be toxic and must be quickly converted to cysteine to prevent harmful buildup. A shortage of NADPH during this conversion can lead to disulfide stress, causing cytoskeletal proteins to form disulfide bonds, contract, and detach from the membrane, disrupting cell function and leading to death.

Contrary to the oncogenic implications outlined earlier, the upregulation of SLC7A11 can also induce apoptotic cell death in cancer cells under conditions. For instance, in glucose-deficient glioblastoma, heightened SLC7A11 expression leads to the generation of ROS and oxidative stress through the consumption of intracellular NADPH during the conversion of imported L-cystine to L-cysteine (58). Moreover, the addition of α-ketoglutarate (α-KG), a metabolite derived from glutamate, effectively restored the viability of cancer cells with elevated levels of SLC7A11 during glucose deprivation, indicating a potential role for exported glutamate in promoting cancer cell death (59). Subsequent research corroborated the significance of converting glutamate to α-KG for the survival of cancer cells in the absence of glucose, highlighting the regulatory function of SLC7A11 in modulating the metabolic adaptability of cancer cells (60). Cancer cells exhibiting elevated levels of SLC7A11 expression demonstrate a reliance on glucose metabolism, while cells with reduced SLC7A11 expression show heightened oxidative phosphorylation (OXPHOS) activity, as observed in lung cancer cell lines. This shift in metabolic preferences has been further validated in specific cell lines, such as A549 and H1299, where SLC7A11 knockdown led to an increase in OXPHOS and a decrease in glycolysis (5). Additionally, this phenomenon has been replicated *in vivo*, within a tumor microenvironment, where the metabolic reprogramming associated with altered SLC7A11 expression was evident (61). We underscore the credibility and reproducibility of the observed metabolic shifts in cancer cells with varying SLC7A11 expression levels (62). Given the limited scope of current experiments, additional research is necessary to explore potential underlying mechanisms or constraints contributing to this paradox. Factors such as the intensity and duration of ROS exposure may offer insight into the observed discrepancies. There appears to exist a toxicity threshold for ROS in the induction of tumor cell death (63). Cell death is only induced at high levels of ROS, while levels below this threshold have been shown to enhance tumor malignancy. This phenomenon has been attributed to the inadequate redox capacity in conditions of limited glucose supply, as well as the abnormal accumulation of cystine or other disulfide molecules in cells with high expression of SLC7A11. This accumulation leads to disulfide stress, ultimately resulting in cell death through a novel form of programmed cell death known as disulfidptosis (64). Subsequent investigations revealed that elevated levels of the SLC7A11 protein unexpectedly heightened the susceptibility of tumor cells to oxidative stressors, leading to increased rates of tumor cell apoptosis. Additionally, *in vivo* experiments demonstrated that heightened SLC7A11 expression facilitated localized tumor growth while impeding tumor migration. In summary, the molecular regulatory mechanism of disulfidptosis involves multiple aspects of cystine uptake, glucose metabolic pathways, disulfide stress, and altered cytoskeletal structure (**Figure 7**).

These findings suggest the potential for inducing distinct forms of cell death in individuals with varying levels of SLC7A11 expression, although the interplay between ferroptosis and disulfidptosis remains an unresolved inquiry.

## Tumor therapeutic strategies targeting SLC7A11

Several compounds have been identified as inhibitors of SLC7A11, including Erastin, IKE, sorafenib, DPI2, SAS, glutamate, and INF-γ. A high-throughput screening of synthetic compounds aimed at identifying substances capable of eliminating tumor cells led to the discovery of erastin, a compound exhibiting RAS-selective activity (65). Notably, erastin was observed to trigger a distinct form of cell death, non-apoptotic in nature. Subsequent investigations delved deeper into its mechanism, revealing that erastin directly inhibits Systems Xc-, thereby depleting GSH levels. The lethality of erastin may primarily stem from its quinazolinone backbone, while other chemical moieties could potentially enhance its inhibitory effects on Systems Xc-. Additionally, erastin targets the mitochondrial voltage-dependent anion system (VDAC) (66). Beyond its direct activation of ferroptosis in the treatment of hepatocellular carcinoma, erastin also potentiates the antitumor effects of certain conventional chemotherapeutic agents in hepatocellular carcinoma cell lines. Furthermore, erastin has the potential to augment the clinical efficacy of PD1/L1 by influencing the polarization of tumor-associated macrophages (TAM) (4). In another study, aspirin was found to elicit a pronounced ferroptosis response in HepG2 and Huh7 cells, an effect that was amplified by the ferroptosis-inducing properties of erastin (67). Given its established role as a classical ferroptosis inducer, erastin has become a benchmark for researchers evaluating novel compounds or assessing the ferroptosis-inducing potential of existing drugs (**Table 2**).

**Table 2:** Small molecule compounds targeting SLC7A11.

Small Molecule Compounds	Target	Cancer	Experimental Models	Reference
erastin	SLC7A11	HCC, GC, CRC	cells, animals	(66, 67)
IKE	SLC7A11	HCC	cells, animals	(68)
DPI2	SLC7A11	HCC	cells, animals	(37)
sorafenib	SLC7A11	HCC	cells, animals, clinical trial	(69–75)
SAS	SLC7A11	NSCLC	cells, animals, clinical trial	(75)
glutamate	SLC7A11	neuroblastoma	cells, animals	(76)
INF-γ	SLC7A11	NSCLC	cells, animals	(77)
HG106	SLC7A11	NSCLC	cells, animals	(78)
Lepadin E/H	SLC7A11	melanoma	cells, animals	(79)
PAB	SLC7A11	glioma	cells, animals	(80)
Ursolic acid	SLC7A11	HCC	cells, animals	(81, 82)
Butyrate sodium	SLC7A11	endometrioma, osteosarcoma	cells, animals	(83)
TPZ	SLC7A11	osteosarcoma	cells, animals	(84)
PZH	SLC7A11	HCC	cells, animals	(85)
levobupivacaine	SLC7A11	GC	cells, animals	(86)
curcumin	SLC7A11	NSCLC	cells, animals	(87)
β-Elemene	SLC7A11	CRC	cells, animals	(88)
Agrimonolide	SLC7A11	ovarian cancer	cells, animals	(89)
Ginkgetin synergized	SLC7A11	NSCLC	cells, animals	(90)
SI	SLC7A11	NSCLC	cells, animals	(91)
vitamin D	SLC7A11	CRC	cells, animals	(92)
Tanshinone IIA	SLC7A11	GC	cells, animals	(93)
Ginsenoside Rh3	SLC7A11	CRC	cells, animals	(94)
Lico A	SLC7A11	HCC	cells, animals	(95)
Talaroconvolutin A	SLC7A11	CRC	cells, animals	(56)
Saikosaponin A	SLC7A11	HCC	cells, animals	(96)

Although erastin demonstrates a potent inhibitory effect on Systems Xc-, its practical application *in vivo* is significantly hindered by its limited water solubility and metabolic instability. To address these challenges, Imidazolidinone (IKE) was developed as a structurally improved derivative of erastin. IKE not only enhances aqueous solubility but also boosts its anticancer properties. Specifically, IKE boasts a solubility that is three times higher than erastin, and it exhibits a remarkable 50-fold reduction in the Lethal Concentration 50 (LC50) for tumor cells, indicating a significantly enhanced antitumor potency (68).

DPI2, a FIN (ferroptosis-inducing compound), which does not inhibit GPx4 activity but specifically inhibits SLC7A11 expression, consumed 90% of the GSH in BjeLR cells compared to the untreated group. The observed effect of DPI2 was comparable to that of erastin, indicating that DPI2 might trigger cellular ferroptosis via a mechanism analogous to that employed by erastin (37).

Sorafenib, a multikinase inhibitor, has gained widespread use in the treatment of clinically advanced hepatocellular carcinoma, offering patients prolonged survival. Previous research attributed sorafenib’s therapeutic effect primarily to its multikinase inhibitory function, which halts cell proliferation, angiogenesis, and promotes tumor cell apoptosis (69, 70). However, recent discoveries indicate that sorafenib’s toxic impact on hepatocellular carcinoma cells relies partly on ferroptosis, rather than apoptosis. This effect isn’t tied to its kinase inhibitory activity but rather to its ability to trigger iron accumulation and lipid peroxidation stress in the cancer cells (71). Notably, depleting stored iron in these cells through iron chelating agents significantly diminishes sorafenib’s cytotoxic effects. Further investigations have uncovered that sorafenib prompts ferroptosis by impeding the activity of the SLC7A11 transporter on the cell membrane. This reduction leads to decreased cystine levels in the cancer cells, subsequently causing insufficient GSH synthesis and a decline in GPX4 activity. Clinically, hepatocellular carcinoma patients treated with sorafenib often develop drug resistance rapidly, influencing their prognosis. Nrf2, Rb, MT-1G, and SIR are involved in regulating the sensitivity to sorafenib-induced ferroptosis through various pathways, contributing to the emergence of drug resistance (66, 72–74). Adopting a fresh perspective, targeting specific inhibitor pathways to stimulate ferroptosis represents a promising strategy to enhance sorafenib’s drug resistance effectively.

Sulfasalazine (SAS) is a long-approved anti-inflammatory drug by the U.S. Food and Drug Administration, serving as a primary therapy for rheumatoid arthritis. SAS promotes ferroptosis through inhibiting the Xc-system, akin to erastin’s mechanism. Nevertheless, SAS is significantly less potent in triggering ferroptosis compared to erastin. Studies have demonstrated SAS’s ability to induce ferroptosis in diverse tumor cells, suggesting its potential as a combinatory treatment with other cancer therapies to enhance overall treatment effectiveness (75).

As an excitatory neurotransmitter that binds to both excitatory neurotransmitter binding sites and Cl^-^dependent cysteine- and cystine-inhibited transporter sites, the Xc-system facilitates the transfer of glutamate out of and cystine in. Glutamate-induced toxicity of cellular ferroptosis is proportional to its ability to inhibit cystine uptake, which is in effect a negative feedback mechanism. Exposure to glutamate leads to a decrease in GSH levels and an accumulation of intracellular peroxides, resulting in oxidative stress and cell death (76).

As talked about previously, tumor-associated immunotherapy studies have shown that CD8^+^ T cells enhance ferroptosis-specific lipid peroxidation in tumor cells. Specifically, INF-γ released by CD8^+^T cells downregulated the expression of SLC7A11 and SLC3A2, leading to a decrease in GSH and an increase in lipid peroxidation. Inhibition of the ferroptosis pathway eliminated the synergistic effect of INF-γ on ferroptosis in tumor cells in both *in vivo* and *in vitro* mouse models (46). And retrospective analysis showed that the expression of SLC7A11 was negatively correlated with CD8^+^ T cell signaling, INF-γ level and prognosis of tumor patients. Combining immunotherapy with SLC7A11 inhibitors produces a powerful therapeutic effect (77).

A powerful SLC7A11 inhibitor, HG106, has been discovered. This inhibitor notably decreases cystine uptake and disrupts intracellular glutathione biosynthesis. Furthermore, HG106 demonstrates targeted cytotoxicity specifically towards KRAS mutant cells. This effect is achieved by amplifying oxidative stress and triggering apoptosis mediated by endoplasmic reticulum (ER) stress. Remarkably, the administration of HG106 in KRAS mutant lung adenocarcinoma (LUAD) models significantly inhibited tumor growth and extended survival rates in multiple preclinical mouse models of lung cancer (78).

Lepadin H, a marine alkaloid, stands out as an effective inducer of ferroptosis. It demonstrates considerable cytotoxicity, stimulates p53 expression, elevates ROS generation and lipid peroxidation, while simultaneously reducing SLC7A11 and GPX4 levels. Additionally, Lepadin H upregulates ACSL4 expression. Remarkably, it exhibits minimal toxicity towards normal organs, highlighting its potential as a transformative ferroptosis inducer (79).

Certain small molecule monomers possess the ability to adjust SLC7A11 activity, thereby influencing intracellular GSH levels and potentially providing therapeutic advantages for treating specific tumors. This hints at the possibility of these monomers acting as innovative ferroptosis inducers by regulating GSH content. One such example is Pseudolaric acid B (PAB), a naturally occurring diterpene acid extracted from Kaempferia roots and bark. Studies have revealed that PAB can trigger ferroptosis in glioma cells by depleting GSH through SLC7A11 inhibition. By suppressing the expression or function of the Xc-system, PAB slows tumor growth *in vivo*, while also inhibiting cancer cell invasion and metastasis (80). This cancer-suppressing effect is primarily attributed to the rapid depletion of GSH caused by SLC7A11 transporter dysfunction, leading to lipid ROS buildup and ferroptosis induction.

Ursolic acid, a pentacyclic triterpenoid derived from traditional plants, significantly boosts ROS accumulation in a hepatocellular model when paired with sorafenib (81). This enhancement is likely due to the downregulation of SLC7A11 expression, causing a drop in intracellular GSH levels and compromising ROS scavenging abilities. As a result, lipid peroxidation occurs, inhibiting ferroptosis and offering notable therapeutic benefits in hepatic cell treatment (82).

Sodium butyrate demonstrates anti-cancer properties by modulating the GSH/GSSG ratio, intracellular ROS levels, and lipid peroxide content, thereby inducing ferroptosis in endometrial cancer cells. It also shows promise in suppressing osteosarcoma growth and metastasis through ferroptosis promotion. Pre-exposure to sodium butyrate intensifies erastin-induced changes in GSH depletion, lipid peroxidation, and mitochondrial morphology in CRC cells. Mechanistically, sodium butyrate down-regulates SLC7A11 transcription by modifying ATF3 expression (83).

Tirapazamine (TPZ), a hypoxic prodrug, is renowned for its antitumor effects in the hypoxic tumor microenvironment. TPZ effectively inhibits all three osteosarcoma cell lines tested. Additionally, TPZ enhances fluorescent staining of ferrous ions while reducing SLC7A11 and GPX4 expression, thus promoting ferroptosis and hindering the proliferation and migration of osteosarcoma cells (84).

Pientzehuang (PZH) demonstrates inhibitory effects on the diethylnitrosamine (DEN)-induced hepatocellular carcinoma (HCC) model in rats. The SLC7A11/GSH/GPX4 axis, associated with the ferroptosis response, is seen as a potential target for PZH in preventing the malignant transition from liver fibrosis to HCC (85).

Levobupivacaine, a renowned local anesthetic, exhibits promising anticancer capabilities. It triggers ferroptosis in gastric cancer cells by manipulating the miR-489–3p/SLC7A11 pathway, thereby hindering cancer cell proliferation (86).

Curcumin, a component derived from turmeric and used in traditional Chinese medicine, has been discovered to cause iron accumulation, GSH depletion, and lipid peroxidation in non-small cell lung cancer (NSCLC) cells. However, suppressing Fer-1 and IREB2—both inhibitors of ferroptosis—substantially diminishes curcumin’s anticancer and ferroptosis-inducing effects in A549 and H1299 cells (87).

β-elemene, a naturally occurring compound, has emerged as a novel inducer of ferroptosis. When combined with cetuximab, it demonstrates enhanced sensitivity towards KRAS-mutant colorectal cancer (CRC) cells by promoting ferroptosis, offering a potential therapeutic approach for such cancers (88).

Agrimonolide, extracted from Lungwort, possesses various biomedical properties, including anticancer activity. In ovarian cancer cell lines A2780 and SKOV-3, Agrimonolide not only restricts proliferation, migration, and invasion in a dose-dependent manner but also initiates apoptosis. Its ferroptosis-inducing effects in ovarian cancer cells are evident from increased ROS, total iron, and Fe^2+^ levels, coupled with reduced expression of ferroptosis markers SLC7A11 and GPX4 (89).

Cisplatin (DDP) stands as a frontline treatment for advanced NSCLC. Ginkgetin has been found to augment DDP’s cytotoxic effects in NSCLC cells by facilitating the accumulation of labile iron and lipid peroxidation. Studies confirm Ginkgetin’s role in mediating ferroptosis through significant reductions in SLC7A11 and GPX4 expression, along with alterations in the GSH/GSSG ratio (90).

Mustardine (SI) has been identified as a potent anti-NSCLC agent, inducing ferroptosis by elevating subferric iron, ROS, and lipid peroxidation. Additionally, SI treatment leads to SLC7A11-dependent downregulation of P53 (91).

Colorectal cancer stem cells (CCSC) significantly impact prognosis, chemotherapy resistance, and treatment outcomes in CRC. Remarkably, Vitamin D administration substantially inhibits CCSC proliferation and reduces tumor spheroid formation *in vitro*. Further analysis reveals that Vitamin D-treated CCSC exhibit elevated ROS levels, decreased cysteine and GSH levels, and thicker mitochondrial membranes, suggesting that SLC7A11 may be a specific target of Vitamin D’s action (92).

Tanshinone IIA, a bioactive compound extracted from Salvia miltiorrhiza, has been found to reduce the stem-like properties of gastric cancer cells. Its mechanism involves increasing lipid peroxidation and decreasing ferroptosis markers in these cells (93).

Ginsenoside Rh3 has proven effective in eliminating colorectal cancer (CRC) cells. It activates Gasdermin D (GSDMD)-dependent pyroptosis and inhibits SLC7A11 through the Stat3/p53/NRF2 pathway, thereby inducing ferroptosis (94).

Licorice chalcone A (Lico A), a key component of the traditional Chinese medicine Glycyrrhiza glabra, is a naturally occurring small molecule with various pharmacological effects. Both *in vivo* and *in vitro* studies have shown that Lico A promotes ferroptosis in hepatocellular carcinoma cells by suppressing SLC7A11 expression. This suppression leads to the inhibition of the GSH-GPX4 pathway and the activation of reactive oxygen species (ROS) (95).

Talaroconvolutin A (TalaA) has emerged as a new inducer of ferroptosis, exhibiting cytotoxicity against colorectal carcinoma cells in a dose- and time-dependent manner. TalaA significantly raises ROS levels to a point where ferroptosis is initiated. Additionally, it downregulates SLC7A11 channel protein expression while upregulating ALOX3, further promoting ferroptosis (56).

Multiple studies have also revealed that Saikosaponin A can induce ferroptosis in hepatocellular carcinoma (HCC) cells, both in laboratory settings and in living organisms. Through RNA sequencing analysis, it has been determined that Saikosaponin A primarily affects the glutathione metabolic pathway and suppresses the expression of the cystine transporter protein SLC7A11. Furthermore, Saikosaponin A has been observed to increase intracellular malondialdehyde (MDA) and iron levels while decreasing reduced glutathione levels in HCC cells. Interestingly, Deferoxamine (DFO), Fer-1, and GSH can reduce the cytotoxic effects of Saikosaponin A, while Z-VAD-FMK is ineffective in preventing Saikosaponin A-induced cell death in HCC (96).

## Conclusions

SLC7A11, as the active subunit of the amino acid transporter on cell membranes, plays a wide range of biological functions in organisms. The regulation of SLC7A11 is affected by multiple dimensions, and also abnormal regulation of SLC7A11 leads to malignant tumors related to proliferation, invasion, metastasis and drug resistance. Given that SLC7A11 is often aberrantly expressed by tissues in tumors, it is expected to be an important biomarker for the diagnosis and prognosis of a wide range of tumors. SLC7A11 presents itself as a viable target for tumor therapy, with two primary strategies currently under consideration. The first involves the development of direct inhibitors targeting SLC7A11 to impede cystine uptake in cancer cells, thereby diminishing intracellular GSH levels and inducing cancer cell ferroptosis. The second strategy entails the utilization of inhibitors targeting glucose transporter proteins or glutaminase in the treatment of tumors overexpressing SLC7A11. This approach capitalizes on the heightened vulnerability of SLC7A11-overexpressing tumors to glucose and glutamine deprivation, ultimately leading to tumor cell death. Nevertheless, the efficacy of the therapeutic strategy is hindered by the inadequate induction of ferroptosis caused by SLC7A11 inhibitors in the presence of intracellular cysteine. Recent studies have demonstrated that combining highly specific SLC7A11 inhibitors with immune checkpoint inhibitors PD1/PDL1 may enhance therapeutic efficacy.

## Prospects and perspectives

SLC7A11 appears to represent a potential Achilles heel for tumor targeting; however, several challenges still need to be addressed. First, most of the treatments for tumors based on SLC7A11 affecting ferroptosis are preclinical studies that are not yet supported by sufficient clinical evidence. Second, the determination of how to define a baseline SLC7A11 expression level is a critical core of tumor treatment strategies based on SLC7A11 expression levels. Differences in the expression levels of SLC7A11 in different tumor tissues, and differences in the expression levels of SLC7A11 between different patients may also be closely related to the temporal, spatial, and individual heterogeneity of tumor tissues. Finally, how to achieve tissue- and cell-targeted delivery of SLC7A11 to maximize the efficacy of the drug to minimize its toxic side effects.

## Author contributions

YJ: Data curation, Methodology, Writing – original draft. MS: Funding acquisition, Resources, Supervision, Validation, Writing – review & editing.
